# Supplementary material for: Improvement of morphophysiological and anatomical attributes of plants under abiotic stress conditions using plant growth-promoting bacteria and safety treatments
Source: PeerJ. 2024 Apr 30;12:e17286. doi: 10.7717/peerj.17286 (PMC11067897; doi:10.7717/peerj.17286)
Supplement: Supplemental Information 1 — Technical changes response [file peerj-12-17286-s001.docx]

***Dear Peer staff***

Thanks so much for your efforts during our review.

Please find below our response to the requested Technical changes.

1. **Figure Permissions**

***Response***:

I confirm that the plant graphic in the center of the figure is created by co-author prof Khaled Abdelaal.
